# Sex differences in schizophrenia-spectrum diagnoses: results from a 30-year health record registry

**DOI:** 10.1007/s00737-023-01371-8

**Published:** 2023-09-20

**Authors:** Maria Ferrara, Eleonora Maria Alfonsina Curtarello, Elisabetta Gentili, Ilaria Domenicano, Ludovica Vecchioni, Riccardo Zese, Marco Alberti, Giorgia Franchini, Cristina Sorio, Lorenzo Benini, Julian Little, Paola Carozza, Paola Dazzan, Luigi Grassi

**Affiliations:** 1 Institute of Psychiatry, Department of Neuroscience and Rehabilitation, University of Ferrara, Ferrara, Italy; 2 Integrated Department of Mental Health and Pathological Addictions, Ferrara, Italy; 3 Department of Psychiatry, Yale School of Medicine, New Haven, CT USA; 4 Department of Engineering, University of Ferrara, Ferrara, Italy; 5 Department of Mathematics, University of Modena and Reggio Emilia, Modena, Italy; 6 School of Epidemiology and Public Health Faculty of Medicine, University of Ottawa, Ottawa, Canada; 7 Department of Psychological Medicine, Institute of Psychiatry, Psychology and Neuroscience, King’s College London, London, UK; 8 National Institute for Health and Care Research (NIHR) Maudsley Biomedical Research Centre (BRC), London, UK

**Keywords:** Psychosis, Women, Precision medicine, Schizophrenia spectrum disorders, Early detection

## Abstract

This study investigated sociodemographic and clinical differences between the sexes in individuals affected by schizophrenia-spectrum disorders (SSD) who accessed outpatient mental health services. Within a retrospective cohort of 45,361 outpatients receiving care in Ferrara (Italy) from 1991 to 2021, those with a SSD diagnosis were compared between the sexes for sociodemographic and clinical characteristics before and after the index date (when the ICD-9: 295.*diagnosis was first recorded) to assess early trajectory, age and type of diagnosis, and severity of illness indicated by medication use, hospitalization, and duration of psychiatric care. Predictors of discharge were also investigated. Among 2439 patients, 1191 were women (48.8%). Compared to men, women were significantly older at first visit (43.7 vs. 36.8 years) and at index date (47.8 vs. 40.6) with peak frequency at age 48 (vs. 30). The most frequent last diagnosis recorded before the index date was delusional disorder (27.7%) or personality disorder (24.3%) in men and depression (24%) and delusional disorder (30.1%) in women. After the index date, long-acting antipsychotics and clozapine were more frequently prescribed to men (46.5% vs. 36.3%; 13.2% vs. 9.4%, *p* < 0.05) and mood stabilizers and antidepressants to women (24.3% vs. 21.1%; 50.1% vs. 35.5%; *p* < 0.05). Women had fewer involuntary admissions (10.1% vs. 13.6%) and were more likely to be discharged as the time under care increased (*p* = 0.009). After adjusting for covariates, sex was not a significant predictor of discharge. Our study confirmed that sex differences exist in clinical and sociodemographic characteristics of outpatients with SSD and that gender considerations might influence the rapidity of diagnosis and medications prescribed. These findings highlight the need to implement a women-tailored approach in specialist care programs for psychoses.

**Supplementary Information:**

The online version contains supplementary material available at 10.1007/s00737-023-01371-8.

## Introduction

Over the last decade, an increasing number of studies have highlighted marked differences between the sexes in the presentation of mental health disorders, including those pertaining to the schizophrenia spectrum disorders (SSD) (Ochoa et al. 2012; Petkari et al. 2017; Riecher-Rossler et al. 2018; Brand et al. 2022). Sex differences in SSD are associated with epidemiological findings, clinical findings, and poorer response to psychotropic medications in men compared to women (Carter et al. 2022; Grigoriadis and Seeman 2002; Sommer et al. 2020). First, compared to men, who typically have their SSD onset in late adolescence-early adulthood, women have a second peak SSD onset in their 50s (Hafner et al. 1994; Häfner and an der Heiden 1997; Cotton et al. 2009; Ochoa et al. 2012; Jongsma et al. 2019). Indeed, in the UK, clinical services dedicated to first-episode psychosis have recently been expanded to provide care to individuals up to the age of 65 years, in order to offer their care to a higher number of women, who otherwise would not have received appropriate care (Jagger et al. 2020; Ferrara and Srihari 2021). Risk factors for SSD also differ between the sexes: women are exposed to the potentially protective role of endogenous estradiol across the life span but more vulnerable than men to the effects of other sexual hormones (Seeman 2000;Riecher-Rössler 2019; Culbert et al. 2022); thus, vulnerability increases during specific periods of major hormonal changes, such as the peri-partum and the menopause (Ochoa et al. 2012; Brand et al. 2021; Sommer et al. 2023). Men often present psychosis with comorbid cannabis use, and they also tend to have negative symptoms, paranoia, aggressivity, impaired cognition, and disorganized behavior, whereas women more commonly present comorbid mood symptoms, with preserved cognition and role functioning (Altamura et al. 2014; Riecher-Rössler 2019; Cowell et al. 1996; Galderisi et al. 2012). As many authors have pointed out, this “atypical”/not-standard presentation of psychosis in women put them at risk of being overlooked, misdiagnosed or, at worst, dismissed as being judged “too fit to be psychotic” (Ferrari et al. 2018; Sommer et al. 2020; Ferrara and Srihari 2021; Mazza et al. 2021). Given these observations, it is possible that efforts aimed at early detection of the first manifestations of schizophrenia may be less effective in women, with consequent diagnostic delay, longer duration of untreated psychosis, and therefore, sub-optimal outcome (Sommer et al. 2020; Ferrara and Srihari 2021). Moreover, women may have a higher risk of adverse pharmacological effects, since only a minority of current medications for schizophrenia have been evaluated in females. Furthermore, prescription of medications in women should take into account their age in relation to reproductive capacity and potential pregnancy planning and the teratogenic effect of certain medications (Santos-Casado and García-Avello 2019). Finally, given the multiple roles often associated with the female gender in western societies, women might experience difficulties accessing care due to lack of resources, time, and a less flexible schedule.

A sex- and gender-oriented approach is thus needed to reduce chronicity, especially for conditions like SSD that cause great disability and suffering for affected individuals and their families, as well as premature death, and result in high direct and indirect health care costs (Christensen et al. 2020). Despite this evidence, guidelines for the comprehensive treatment of schizophrenia in women are lacking (Fernando et al. 2020; Seeman 2020; Ferrara and Srihari 2021).

One of the reasons behind the lack of sex and gender sensitive approaches to schizophrenia is that current literature provides limited evidence on gender differences throughout the full course of schizophrenia. More complete evidence could support the provision of tailored recommendations for the diagnosis and treatment of SSD in women (Beery and Zucker 2011; Seeman 2018; Bölte et al. 2023). Thus, there is a need for evidence from longitudinal data on large samples of individuals with schizophrenia, assessing their pathways to and through care, illness trajectory, treatment offered, and clinical outcomes. One of the first such studies was based on the Finnish national registry (Sommer et al. 2020). This study confirmed that differences exist between the sexes in SSD—women, compared to men, were diagnosed later and were less frequently prescribed clozapine and long-acting antipsychotics, and they died more often of cancer (Sommer et al. 2020). However, this study included individuals selected on the basis of a SSD diagnosis received during an inpatient hospitalization, but it is known from other studies that not all individuals have SSD diagnosed in hospital (Vanasse et al. 2012), and an in-patient hospital diagnosis of SSD is made less frequently in women than men (Seeman 2019; Ferrara and Srihari 2020); thus, it is likely that the proportion of women with SSD was underestimated in the Finnish study (Seeman 2019). Data originating from a large epidemiological outpatient community-based registry, such as the Italian FEPSY data registry (Ferrara et al. 2023), could provide a valuable additional source of information about sex differences in SSD, regardless of admission to inpatient units.

The present study is aimed at investigating the sociodemographic and clinical differences between sexes in individuals affected by SSD who accessed outpatient mental health services in Ferrara, Northern Italy, during a period of 30 years, to assess early trajectory, age and type of diagnosis, and severity of illness expressed by medication use, hospitalization, and duration of treatment. Predictors of discharge from the service were also investigated.

## Methods

### Participants and study design

The FEPSY cohort included all individuals who had access to the services provided by the Integrated Department of Mental Health and Pathological Addiction (DAISMDP) between 1991 and 2021 in the province of Ferrara. The Ferrara Province covers a catchment area of 2630 km^2^ and 342,000 inhabitants. The DAISMDP offers mental health care to the entire province under the national universal healthcare system.

Sociodemographic and clinical data are routinely entered by clinicians in an Electronic Health Record (EHR) system. For the study period, these data were anonymized and assembled for research purposes in the FEPSY database, as documented by Ferrara et al. 2023 (Ferrara et al. 2023). Thus, the present analysis is based on 3,861,432 records on 46,222 individuals in the FEPSY database (Supplementary Table 1).

All patients who received a diagnosis of schizophrenia-spectrum disorder (SSD), consisting of either schizophrenia or schizoaffective disorder (ICD-9: 295.*), regardless of age, were selected and included in this study. The date on which the patient received the 295.* diagnosis was defined as the index date.

The study was approved by the Local Ethical Committee (CE-AVEC) (Protocol Number 197/2018). All data were handled in accordance with the General Data Protection Regulation (EU) 2016/679. According to the Data Protection agreement by the Italian Guarantor for the Protection of Personal Data (Number 85, March 1, 2012), written informed consent was not required since this study used anonymized registry data (Ferrara et al. 2023).

### Variables and assessment

The primary objective was to assess sociodemographic and clinical differences between the sexes at service presentation as well as illness trajectory. To do so, sociodemographic data (sex, age at first visit, age at index date, married status, living condition, and catchment area) and clinical information before and after the index date (psychiatric diagnoses, medication prescribed, psychiatric inpatient hospitalization—number, duration, voluntary vs. compulsory, and time under the care of psychiatric services) were analyzed. Psychotropic medications were classified based on the regional code (Supplementary Table 2). They were also categorized as oral vs. long-acting injected antipsychotic (LAI). Clozapine was analyzed separately because it is typically used specifically for treatment resistant schizophrenia.

The secondary objective was to assess potential predictors of discharge from the service. Duration of psychiatric care was calculated as the time interval in days between the first and the last psychiatric visit up until February 22, 2023 (day of the last update). Time to discharge (time under care of psychiatric services) was calculated as the time interval in years between the SSD diagnosis and discharge from services.

### Statistical analysis

For continuous data, descriptive statistics are presented as means with standard deviations (SD). Medians with ranges (min–max) are also provided for highly skewed distributions. Differences between the two sexes in sociodemographic characteristics, prevalence of comorbidities, and medication use were analyzed using bivariate analysis. Chi-square analyses were used for categorical variables, while Mann Whitney *U* tests and *t*-test were used for continuous variables. Kaplan-Meier survival curves were used to analyze differences in time to discharge (time under care), by sex. Time under care was calculated as duration, in years, between date of first 295* diagnosis and date of discharge. Patients still in care at the last update (02/22/2023) were censored (Klein and Moeschberger 2003). Log rank test was used to test differences between groups. A multivariable model was fit using proportional hazards regression to assess whether sex was associated with time to discharge. Potential covariates were controlled by adjusting for other variables included in the study. Hazard ratios (HR) and related confidence intervals (CI) were reported. All analyses were performed in R (version 4.2.2). A *p* value < 0.05 was deemed to be statistically significant.

## Results

The FEPSY dataset included a total of 45,361 individuals with a psychiatric diagnosis and is detailed in Supplementary Table 1. Within FEPSY, a total of 2439 patients with a SSD diagnosis were identified for this study, of whom 1191 were women (48.8%) (Table 1).

**Table 1:** Sociodemographic and clinical characteristics of the study population (*N* = 2439)

Characteristics	Males	Females	*p* value
	(*N* = 1248)	(*N* = 1191)	
*Sociodemographic characteristics*			
Age at first visit, years Mean ± SD (median; min–max)	36.9 ± 13.93 (34; 13–85)	43.67 ± 15.08 (41; 15–95)	< 0.05^a^
Age at index date (295.* diagnosis), years Mean ± SD (median; min–max)	40.62 ± 14.55 (38; 13–85)	47.80 ± 15.24 (47; 18–95)	< 0.05^a^
Nationality (born in Italy/outside Italy), *N* (%)			ns^c^
In Italy	1148 (91.99%)	1098 (92.19%)	
Abroad	91 (7.29%)	88 (7.39%)	
Missing	9 (0.72%)	5 (0.42%)	
Residence in Ferrara, *N* (%)			< 0.05^c^
Yes	428 (34.29%)	478 (40.13%)	
No	781 (62.58%)	669 (56.17%)	
Missing	39 (3.13%)	44 (3.69%)	
Marital status, *N* (%)			< 0.05^c^
Single	770 (61.70%)	401 (33.67%)	
Married or living with partner	136 (10.90%)	359 (30.14%)	
Separated/divorced/widowed	94 (7.53%)	198 (16.62%)	
Missing	248 (19.87%)	233 (19.56%)	
Education level, *N* (%)			< 0.05^c^
Illiterate	95 (7.61%)	138 (11.59%)	
Literate	207 (16.59%)	165 (13.85%)	
Primary school	200 (16.03%)	176 (14.78%)	
Middle school	232 (18.59%)	171 (14.36%)	
High school	146 (11.70%)	149 (12.51%)	
University	34 (2.72%)	32 (2,69%)	
Missing	334 (26.76%)	360 (30.23%)	
*Clinical characteristics*			
Number of hospitalizations before index date Mean ± SD (median; min–max)	1.04 ± 2.90 (0; 0–42)	0.98 ± 2.91 (0; 0–39)	ns^b^
Number of hospitalizations after index date Mean ± SD (median; min–max)	2.40 ± 5.35 (0; 0–60)	2.28 ± 6.93 (0; 0–108)	< 0.05^b^
Duration of hospitalizations before index date, days Mean ± SD (median; min–max)	15.89 ± 62.14 (0; 0–1780)	13.59 ± 36.64 (0; 0–430)	ns^b^
Duration of hospitalizations after index date, days Mean ± SD (median; min–max)	36.02 ± 84.14 (0; 0–966)	35.73 ± 92.61 (0; 0–1120)	ns^b^
Proportion (%) of patients who had one or more compulsory admissions before index date	5.53%	4.03%	ns^c^
Proportion (%) of patients who had one or more compulsory admissions after index date	13.62%	10.08%	< 0.05^c^
Delay in diagnosis^d^, days Mean ± SD (median; min–max)	1405.12 ± 2.090.93 (44; 0–9986)	1512.01 ± 2.207.48 (57; 0–9994)	ns^b^

^a^*t*-test; ^b^Mann-Whitney *U*; ^c^chi-squared test; note: ^d^delay in diagnosis: time difference between enrollment date and index date

**Fig. 1:**
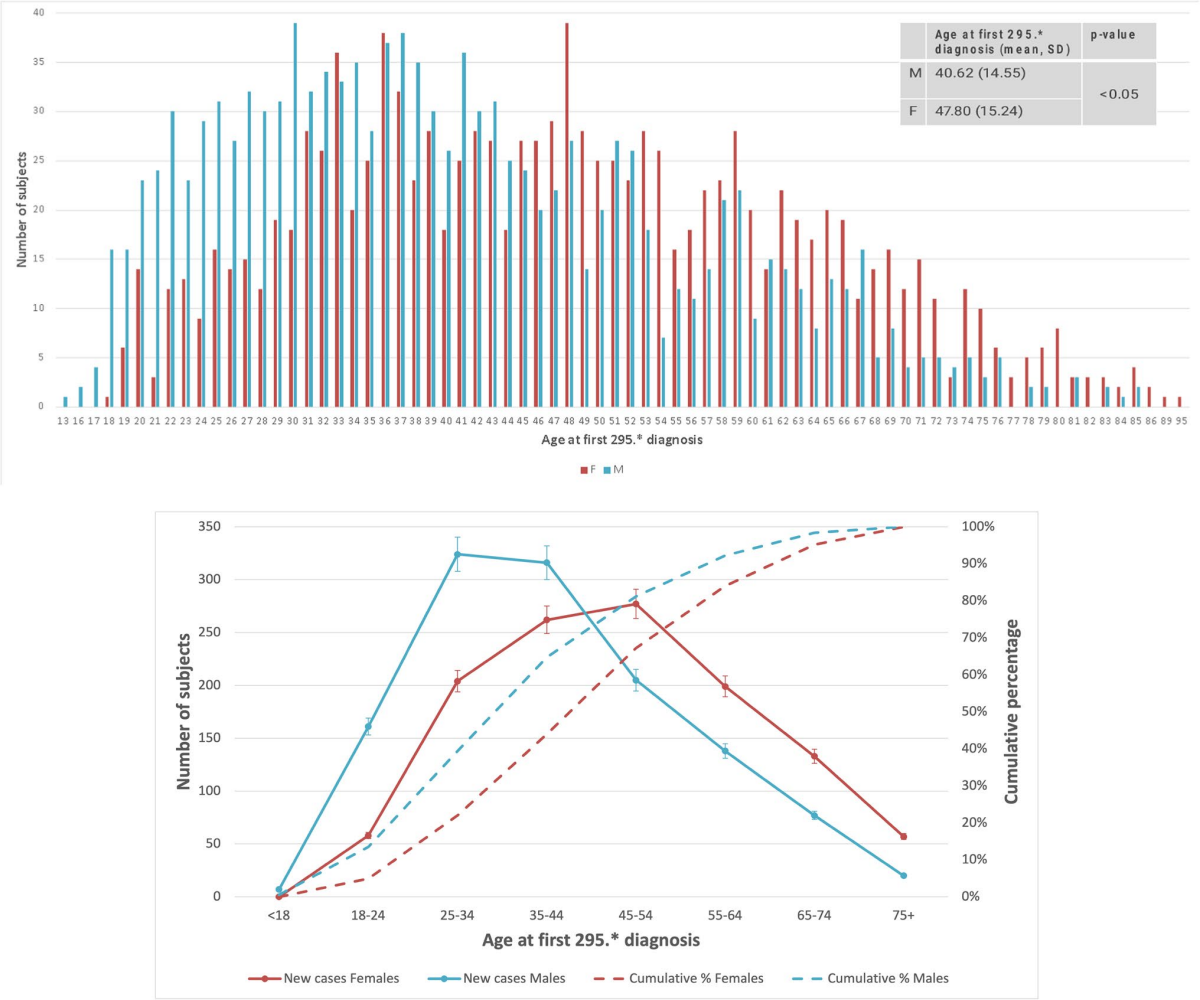
Upper panel: age at index date (first ICD-9 295.* diagnosis): men are represented in blue, women in red. Lower panel: new and cumulative cases by sex

Compared to men, women were on average significantly older at first visit to a mental health service (43.7 [SD 15.1] vs. 36.8 [SD 13.9] *p* < 0.05) and at time of first diagnosis of SSD (47.8 [SD 15.2] vs. 40.6 [SD 14.6]); *p* < 0.05), with peak frequency at age 48 years old (vs. 30) (Fig. 1).

Women were significantly more likely to be resident in the city of Ferrara, be married, and to have a high school degree (*p* < 0.05).

**Fig. 2:**
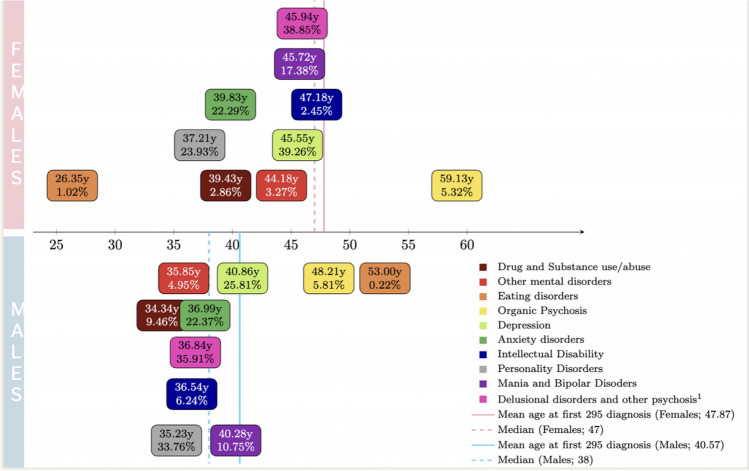
Mean age at first psychiatric diagnosis (*other than 295.*). Women in the upper section vs. men in the lower section

Paranoid schizophrenia was the most prevalent diagnosis in both sexes (32.13% in men, 26.95% in women), while schizoaffective disorder was almost twice as prevalent in women compared to men (22.84% vs. 12.98%). Men and women differed significantly in terms of the last diagnosis received before the index date. In men, this was most frequently delusional disorder (27.7%) or personality disorder (24.3%), while women’s last diagnoses were most frequently depression (24%) and delusional disorder (30.1%) (*p* < 0.05). The mean age at first diagnosis of any mental disorder (other than 295.*) was also different between the sexes, with women being diagnosed at an older age compared to men for all diagnostic categories except for eating disorders (Fig. 2).

Before the index date, 92.3% of women (vs. 91.6% of men, *p* = ns) were not prescribed psychotropic medications. If pharmacological treatment was prescribed, both women and men received mainly oral antipsychotics (80.4% vs. 88.6%) or anxiolytics (66.3% vs. 76.2%). LAIs were prescribed more frequently to men than women (37.1% vs. 33.7%), as well as mood stabilizers (17.1% vs. 14.1%).

After the index date, both sexes were treated mainly with oral antipsychotics (90.3% and 86.1%). LAIs were most frequently prescribed to men (46.5% vs. 36.3%; *p* < 0.05), as was clozapine (13.2% vs. 9.4%, *p* < 0.05). Mood stabilizers were more often prescribed to women (24.3% vs. 21.1%; *p* < 0.05), as were antidepressants (50.1% vs. 35.5%; *p* < 0.05), and anxiolytics/hypnotics (75.9% vs. 73.3%; *p* < 0.05). After the index date, women were less likely to be hospitalized (2.28 vs. 2.40, *p* < 0.05) and to be admitted to hospital on an involuntary basis.

**Fig. 3:**
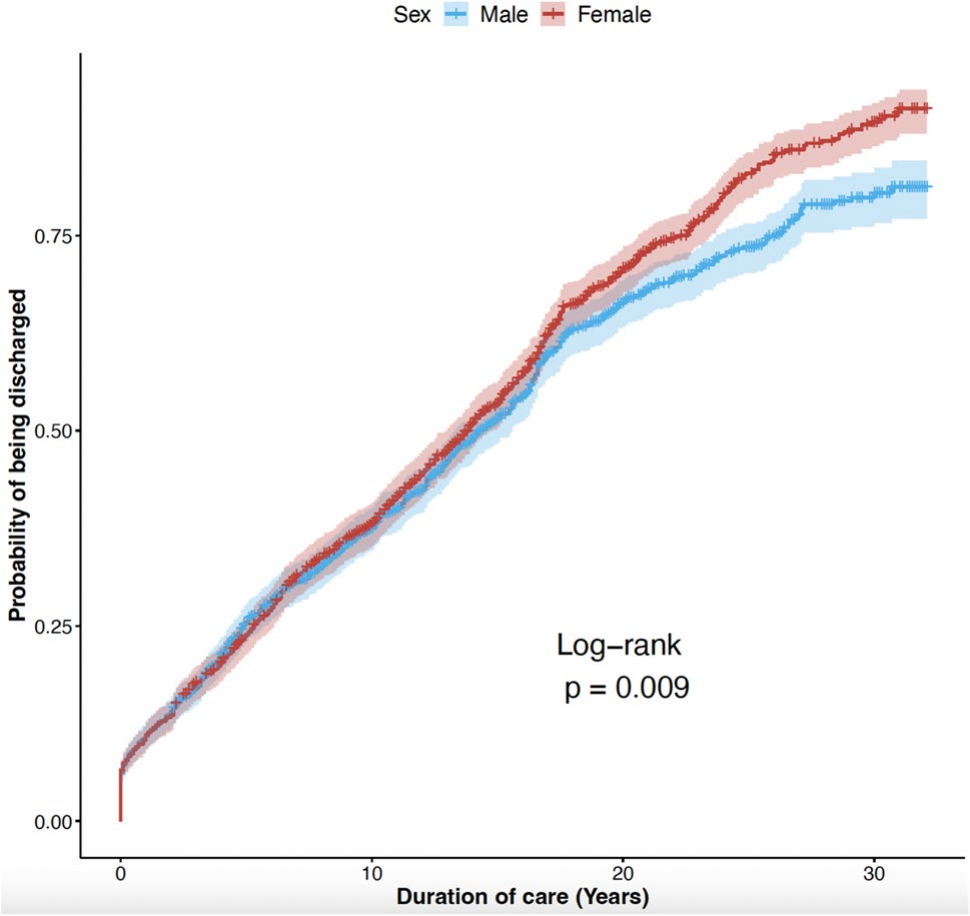
Probability of being discharged from the community psychiatric service, stratified by sex without adjusting for covariates

Although, during the first 10 years of treatment, both sexes had the same probability of being discharged, significant differences emerged over a longer period (Fig. 3). Men were at lower risk of being discharged after 20 or 30 years after their first contact with the community mental health center (CMHC) (log-rank, *p* = 0.009). In unadjusted proportional hazard regression, men were about 12% less likely to be discharged (HR = 0.88; *p* = 0.009). However, after adjusting for sociodemographic and clinical characteristics, sex appeared to no longer influence time to discharge. In the adjusted model, patients who were born in Italy (HR = 0.30, *p* < 0.001), resident in Ferrara (HR = 0.81, *p* < 0.001), or separated (HR = 0.77, *p* = 0.003) were less likely to be discharged from the CMHC. No difference was found between patients who were treated with mood stabilizers vs. those who were not, and those who were prescribed antidepressants vs. those who were not. By contrast, patients were less likely to be discharged if that had been prescribed clozapine (hazard ratio 0.25, *p* < 0.001), oral antipsychotics (0.43, *p* < 0.001) or LAI (0.35, *p* < 0.001) (Table 2).

**Table 2:** Adjusted hazard ratios for discharge. Abbreviations: *CI*, confidence interval; *p*, *p* value

	Risk of discharge (*n* = 1886) Hazard ratio		
Predictors	Estimates	CI	*p*
Sex [M]	1.06	0.94–1.20	0.336
Age at first visit	1.02	1.02–1.03	< 0.001
Age at first visit < 25	1.12	0.90–1.39	0.307
Born in Italy [yes]	0.30	0.24–0.38	< 0.001
Residence in Ferrara [yes]	0.81	0.72–0.91	< 0.001
Separated [vs. married]	0.77	0.64–0.91	0.003
Delay to index date	1.00	1.00–1.00	< 0.001
Number of hospitalizations before index date	1.03	1.00–1.06	0.057
Number of hospitalizations after index date	0.97	0.95–0.99	0.003
Past or current treatment with:			
Mood stabilizer	0.95	0.75–1.20	0.665
Antidepressant	0.87	0.73–1.04	0.121
Clozapine	0.25	0.17–0.35	< 0.001
Oral antipsychotic	0.43	0.37–0.50	< 0.001
Long-acting injectable antipsychotic	0.35	0.28–0.42	< 0.001

## Discussion

This 30 year-long health record registry supports evidence for differences between the two sexes in the clinical and sociodemographic characteristics of individuals with a diagnosis of schizophrenia-spectrum disorders. To our knowledge, this is the first study describing the course of illness before and after a schizophrenia diagnosis from a sex-specific perspective within an outpatient community psychiatry cohort.

The main findings of our study confirm that women are more likely to be diagnosed in their late 40s and to show less compromised functioning both at presentation and in the long term: compared to men, they are more likely to be married, with a higher degree of education, less likely to have a history of psychiatric hospitalizations, less likely to be prescribed LAIs and clozapine, and less likely to be still in care 10 years after their first visit. The finding about marital status is consistent with data from the general population: in Italy, in the 15–64 age group, the proportions of married and unmarried men are almost equivalent (49.0% vs. 47.7%) while the proportion of married women is higher than the proportion unmarried (55% vs. 39% (Istituto Nazionale di Statistica ISTAT 2018); moreover, men tend to marry at an older age compared to women. Consistent with the general population, women appeared to have a higher education (Istituto Nazionale di Statistica Istat 2021). Our findings are consistent with the largest incidence study conducted in Europe (Jongsma et al. 2018) that found the incidence of treated SSD in women peaks in their 50s compared to men in their 20s and that most individuals who had their FEP after the age of 35 were women (Taylor et al. 2023). Our findings are also consistent with the widely accepted observation that women have a less severe clinical profile at presentation, which can in turn result in delayed diagnosis (Ferrara and Srihari 2021). Indeed, in our sample, before the index date, women had most often been diagnosed with depression, whereas men had most often been diagnosed with personality disorders. Contrary to the report by Sommer et al. (2020), the delay in receiving an SSD diagnosis was not significantly different between the two sexes. However, since this delay was almost 4 years, the delay urges some thinking about clinicians’ ability to identify SSD in women. In the last 20 years, a lot of resources have been devoted to the prompt identification of young individuals with a first episode of psychosis (Patel et al. 2007). What is still missing though is a strategy for the rapid identification of early signs of SSD that may differ by sex and gender (Seeman 2020;Ferrara and Srihari 2021). This could for example involve more training for professionals about the specific presentation of SSD in women (less adherent to the conventional representation of SSD), more attention to female-specific risk factors such as the peripartum period, or the vulnerability conferred by a history of violence or abuse (Seeman 2018), and major hormonal changes (Sommer et al. 2023).

Pharmacological treatment also differed between the sexes. Specifically, LAIs and clozapine were more frequently prescribed to men. This finding is consistent with several other studies that have shown that women are less often prescribed LAI (Medrano et al. 2018). While investigating the rationale behind supporting this prescription patterns was beyond the scope of our work, we propose some hypotheses. First, it is possible that men were expected to be less adherent to medications and thus a long acting formulation was more likely to be offered, even though evidence in this direction is lacking (Castberg et al. 2009). Second, clozapine, a drug that is generally under prescribed for clinical and logistic reasons (e.g., the frequent blood monitoring, and the risk of severe adverse effects (Joober and Boksa 2010)), seems to be less likely to be prescribed to women (Wellesley Wesley et al. 2021; Sommer et al. 2020), who are therefore at higher risk of being deprived of an effective treatment. It might also be possible that concerns about weight gain associated with perceived societal expectations on how a female body should appear might discourage women from accepting treatment with clozapine (Ferrara and Srihari 2020; Nielsen 2021).

Another important finding is that, compared to men, after the first 10 years under the care of psychiatric service, women are more likely to be discharged from the CMHC; however, this difference did not hold after adjusting for covariates. This is consistent with two other studies that found that both sexes had similar outcomes after 10–13 years of treatment (Ayesa-Arriola et al. 2020;Mayston et al. 2020). However, in the longer term, men seem to spend longer time under the care of services. Usually, longer time under care points to a more chronic phenotype. However, as Seeman has pointed out, over the past decades, the definition of what represents a good or bad outcome in SSD has dramatically changed (Seeman 2019), with more attention focused on personal and functional recovery, patients’ engagement, and input into definition and ranking of outcomes (Srihari et al. 2016). More information is needed to understand further these trajectories of care by sex and gender.

### Strengths

A strength of this study is that, unlike RCTs, there were no restrictions on inclusion criteria, as all patients are automatically registered in the EHR as soon as they enter care regardless of comorbidity, severity of illness, and referrals, thus providing real-life data. This makes this cohort comparable to similar EHR-based studies. Another strength is the sample size and the 30-year-long period of observation which allowed for a comprehensive evaluation of both the presentation and outcome of SSD in the short and long term.

### Limitations

The results of this study should be interpreted in light of some limitations. First, the retrospective design of the study is prone to missing information and reliability problems. This particularly affected some variables, including marital status, educational level, and employment status which are recorded at admission but not regularly updated. Moreover, information regarding medication for physical illness (e.g., diabetes, hypertension, and obesity) prescribed by general practitioners unfortunately were not available in FEPSY. However, our main findings are consistent with previous literature, indicating a reasonable degree of reliability in our measures. Second, the cohort refers to a limited catchment area, and some results might not be generalizable to areas or countries with very different socio-economic contexts and network of care. However, we believe that the universal health care offered in Italy is comparable to that of most other European countries, making our findings representative of at least this part of the western world. Another limitation is that this cohort relies on a diagnosis based on clinical assessment, which might favor the diagnosis of SSD in men, who present more often than women with a conventional manifestation of schizophrenia. Moreover, some information that could influence pathways to and through care was not routinely recorded in EHR, such as symptom severity, economic status, comorbid medical conditions, and pregnancies. Finally, the analysis of resource utilization, including number of visits and personnel involved, was beyond the scope of this study; future analysis of this type would inform service planning.

### Future directions

The findings of this study suggest that it may be appropriate to refine First Episode Psychosis (FEP) services, which were originally conceptualized around young people needs regardless of sex. However, most women have their onset later in life and could benefit from a specialist service that include female-tailored care such as individual psychotherapy, parenting training, and liaison with community preventive medicine (e.g., cervical cancer screening). In addition, currently operating FEP services could inform general community mental health centers through joint educational activities, case management, and ad hoc consultations. The evolution of FEP services to accommodate women’s care needs is possible only by involving all relevant stakeholders: female users with lived experience, service providers, and potential referrers such as general practitioners, maternity clinics, women’s shelters, and abuse survivors centers (González-Rodríguez et al. 2020; Ferrara and Srihari 2021; Diaz-Pons et al. 2022).

While local stakeholders identify the resources for community mental health services, possible quality improvement initiatives could be already implemented. For instance, two CMHCs in Barcelona, Spain, are promoting a collaborative multidisciplinary and multispecialty network program that will include the provision of perinatal mental health, a liaison with a medical unit, prevention of suicide risk, interventions on parenting, domestic abuse and sexual exploitation, home-based services, peer support, and occupational therapy (González-Rodríguez et al. 2023).

In conclusion, our 30-year-long record registry confirms that biological sex influences age of onset, pharmacological treatment, risk of hospitalization, and duration of illness in individuals diagnosed with SSD. These findings highlight that both sex and gender can influence the trajectory of SSD and therefore its outcomes. There is the need to implement a tailored sex-approach in specialized programs for psychosis. Implementation studies are needed to explore the efficacy and effectiveness of sex-tailored approaches for diagnosing and treating SSD in women.

### Supplementary information

ESM 1 (DOCX 19 kb)
