# Supplementary material for: Sex differences in schizophrenia-spectrum diagnoses: results from a 30-year health record registry
Source: Arch Womens Ment Health. 2023 Sep 20;27(1):11–20. doi: 10.1007/s00737-023-01371-8 (PMC10791808; doi:10.1007/s00737-023-01371-8)
Supplement: Supplementary file 1 — (DOCX 19 kb) [file 737_2023_1371_MOESM1_ESM.docx]

**Supplementary Table 1.** Overall sample FEPSY: comparison between individuals with and without the SSD diagnosis.

|  | **With SSD [295.*] (N=2,439)** | **Without SSD [295.*]**  **(N= 42,922 )** | **p-value** |
| --- | --- | --- | --- |
| **Gender** |  |  | **<0,05 ^c^** |
| F | 1191 (48,83%) | 26373 (61,44%) |  |
| M | 1248 (51,17%) | 16549 (38,56%) |  |
| **Age at first visit** | 40,15 ±14,90 (37; 13-95) | 50,34 ±18,96 (49; 1-109) | **<0,05 ^a^** |
| **Residence in Ferrara** |  |  | **ns ^c^** |
| No | 1450 (59,45%) | 25901 (60,34%) |  |
| Yes | 906 (37,15%) | 15834 (36,89%) |  |
| Missing | 83 (3,40%) | 1187 (2,77%) |  |
| **Marital Status** |  |  | **<0,05 ^c^** |
| Single | 1171 (48,01%) | 9878 (23,01%) |  |
| Married / with Partner | 495 (20,30%) | 15882 (37,00%) |  |
| Separated/Divorced/Widowed | 292 (11,97%) | 6867 (16,00%) |  |
| Missing | 481 (19,72%) | 10295 (23,99%) |  |
| **Education level** |  |  | **<0,05 ^c^** |
| Illiterate | 233 (9,55%) | 4083 (9,51%) |  |
| Literate | 372 (15,25%) | 4814 (11,22%) |  |
| Primary school | 376 (15,42%) | 5333 (12,42%) |  |
| Middle school | 403 (16,52%) | 5446 (12,69%) |  |
| High school | 295 (12,10%) | 5967 (13,90%) |  |
| University | 66 (2,71%) | 1801 (4,20%) |  |
| Missing | 694 (28,45%) | 15478 (36,06%) |  |
| **Catchment Area** |  |  | **<0,05 ^c^** |
| Ferrara | 934 (38,29%) | 16459 (38,35%) |  |
| Codigoro | 523 (21,44%) | 6556 (15,27%) |  |
| Portomaggiore | 258 (10,58%) | 4989 (11,62%) |  |
| Cento | 220 (9,02%) | 5241 (12,21%) |  |
| Copparo | 188 (7,71%) | 4216 (9,82%) |  |
| Missing | 316 (12,96%) | 5461 (12,72%) |  |
| **Treatment Duration (days) (sum of individual charts)** | 4600,24 ±3127,74 (4664; 1-14360) | 1286,93 ±1917,89  (204; 0-13313) | **<0,05 ^b, d^** |
| **Treatment duration (first access -last visit)** | 4995,61 ±3639,27 (5301; 1-11105) | 1023,36 ±2008,15  (35; 1-11099) | **<0,05 ^b, d^** |
| **Treatment Duration (days) (sum of individual therapeutic product)** | 4367,27 ±3037,54 (4447; 1-11006) | 1193,42 ±1820,14  (143; 0-10929) | **<0,05 ^b, d^** |
| **Total duration of hospitalization (days)** | 50,65 ±113,86  (10; 0-2017) | 2,86 ±24,42  (0; 0-2271) | **<0,05 ^b, d^** |

We included **45,361** individuals out of the 46,222 registered in FEPSY in this study. 62 individuals were excluded since it was not possible to retrieve their index date (date of the first 295.* diagnosis); 799 individuals were excluded since they had medical records or products with inconsistent dates (start date > end date).

^a^ t-test

^b^ Mann-Whitney U

^c^ chi-squared test

^d^ Mood’s Median test

**Table 2: Classification of Medications**

The Anatomical Therapeutic Chemical classification was adopted. Medications of interest were sub-divided into 5 groups:

1: Mood stabilizers: carbamazepine, valproic acid, lithium

2: Antipsychotics: including antipsychotics, long-acting formulation, clozapine*

3: Antidepressants

4: Anxiolytics, hypnotics

5: Other medications – including opioids, anesthetics, dementia medication, nootropics, antiepileptics.

* group n#2 originally included lithium that was moved to group n#1

* group n#5 originally included carbamazepine and valproic acid (antiepileptics) that were moved to group n#2
